# Downregulation of LRRC19 Is Associated with Poor Prognosis in Colorectal Cancer

**DOI:** 10.1155/2022/5848823

**Published:** 2022-06-26

**Authors:** Ya-Juan Wang, Man Liu, Hui-Ying Jiang, Yong-Wei Yu

**Affiliations:** ^1^Department of Pathology, Shengzhou People's Hospital, Shengzhou Branch of the First Affiliated Hospital of Zhejiang University, Shengzhou, Zhejiang, China; ^2^Department of Clinical Laboratory, Shengzhou People's Hospital, Shengzhou Branch of the First Affiliated Hospital of Zhejiang University, Shengzhou, Zhejiang, China; ^3^Intensive Care Unit, Shengzhou People's Hospital, Shengzhou Branch of the First Affiliated Hospital of Zhejiang University, Shengzhou, Zhejiang, China; ^4^Intensive Care Unit, The First Affiliated Hospital, Zhejiang University School of Medicine, Hangzhou, China

## Abstract

**Objective:**

Colorectal cancer (CRC) is globally one of the most often diagnosed cancers with high mortality rates. This study aimed to explore novel biomarkers for the diagnosis and prognosis of CRC.

**Methods:**

We collected 4 datasets about CRC in GEO and sought differentially expressed genes (DEGs) with GEO2R. Leucine-rich repeat-containing protein 19 (LRRC19) expression was assessed through the Oncomine and TIMER database analyses, which was further confirmed by qRT-PCR of CRC samples. We used online survival analysis tools (GEPIA, PrognoScan, and Kaplan–Meier plotter) to examine the prognostic value of LRRC19 in CRC and other malignancies. GO and KEGG enrichment analyses were employed to explore the biological functions of LRRC19. Finally, we conducted network prediction by STRING and further validation on the GEPIA to discover other molecules that might interact with LRRC19.

**Results:**

A total of 21 upregulated and 46 downregulated DEGs were identified from the 4 datasets. The TIMER and Oncomine online analyses showed lower mRNA of LRRC19 in CRC tissues compared with adjacent normal tissues, which was validated by qRT-PCR in CRC patient samples. The survival analysis through the GEPIA and PrognoScan websites revealed that low LRRC19 expression was significantly correlated with poor prognosis in CRC patients. The Kaplan–Meier plotter survival analysis indicated that low LRRC19 expression was significantly associated with the disease progression of patients with ovarian cancer, gastric cancer, breast cancer, and lung cancer. The enrichment analysis suggested that low expression of LRRC19 could be involved in the retinol metabolism and the zymogen granule membrane. Through STRING and GEPIA, it was found that LRRC19 is clearly associated with ZCCHC10, MOB3B, IMMP2L, and TRMT11.

**Conclusion:**

LRRC19 mRNA was prominently decreased in human CRC tissues and was significantly associated with shorter survival in CRC patients. LRRC19 might serve as a possible target for early diagnosis and prognosis assessment in CRC.

## 1. Introduction

Among the most commonly diagnosed cancers, colorectal cancer (CRC) ranked third in men and second in women, with an estimated 1.4 million cases and 693,900 deaths in 2012 worldwide [1]. Although investigators have tried hard to uncover the molecular mechanism of occurrence and progression of CRC, it has not been thoroughly illustrated. Therefore, it is necessary to further explore CRC-related genes and pathways, which helps not only to unravel the molecular mechanism of the tumorigenesis and development of CRC but also to guide the development of hopeful diagnostic and prognostic biomarkers and optimal therapeutic strategies [2].

The past few years of research obtained increased tumor biomarkers related to the progression or prognosis of human cancers. A number of above-mentioned biomarkers have been assessed in CRC patients, such as BRAF, KRAS, NRAS, and MMR [3–5]. The gene chip detection technique, which can recognize all genes within the same sample time-point expression information, is generally used to screen differentially expressed genes (DEGs). Publicly available databases, including the Gene Expression Omnibus (GEO) and The Cancer Genome Atlas (TCGA), have stored huge amounts of core microarray data about the relationship between genes and cancers at the gene level [6, 7]. Therefore, a mass of gene expression profiles and prognostic biomarkers have been accumulated regarding CRC. However, as a result of independent sample heterogeneity, the outcomes were different or limited. To address these deficiencies, our study used the method of integrated bioinformatics with expression profiling techniques to recognize steady biomarkers for CRC.

**Figure 1:**
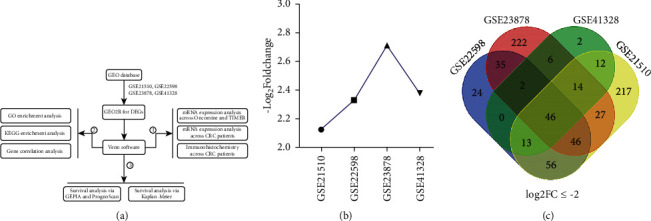
Flowchart and Venn diagram of DEGs. (a) Flowchart. (b) LRRC19 expression level in 4 GEO databases. (c) 46 DEGs downregulated in the four datasets (log2FC ≤ −2). Different colors meant different datasets. The overlapped areas show the number of DEGs among GSE21510, GSE22598, GSE23878, and GSE41328.

We employed four microarray datasets (GSE215108, GSE225989, GSE2387810, and GSE4132811) to screen DEGs in CRC tissues by virtue of the GEO2R tool and Venn diagram software [8–11]. As a result, we found that LRRC19 mRNA was significantly downregulated in CRC samples compared with adjacent normal tissues. Based on the microarray findings, qRT-PCR was performed for further expression validation and to explore the association between the expression level of LRRC19 and the clinicopathological features. Then, a combination of online databases (including GEIPA, UALCAN, and Kaplan–Meier plotter) was further performed to assess the correlation between LRRC19 expression and survival in CRC patients. Meanwhile, the analyses of Gene Ontology (GO) and Kyoto Encyclopedia of Gene and Genome (KEGG) enriched pathways were also conducted for annotation and visualization with LRRC19 potential function. Finally, a network of LRRC19 interactions with other molecules was predicated in the STRING and GEPIA databases (Figure 1(a)).

## 2. Materials and Methods

### 2.1. Data Resource and Description

Original data between CRC tumor and nontumor samples were downloaded from the Gene Expression Omnibus (GEO; https://www.ncbi.nlm.nih.gov/) database, and four gene expression profiles (GSE21510, GSE22598, GSE23878, and GSE41328) were elected [12]. The array data of GSE21510 comprised 104 CRC and 44 adjacent normal tissues [8]. GSE22598 included 17 CRC and 17 adjacent normal tissue samples [9]. GSE23878 consisted of 35GC and 24 adjacent normal tissue samples [10]. Finally, GSE41328 contained 10 CRC and 10 adjacent normal tissue samples [11].

### 2.2. Data Preprocessing of DEGs

The GEO2R (https://www.ncbi.nlm.nih.gov/geo/geo2r/) online analysis tool was used to screen differentially expressed genes (DEGs) between CRC and adjacent normal tissues [7]. The adjusted *P* values were applied to correct the occurrence of false-positive results using the Benjamini and Hochberg false discovery rate method by default [13]. The fold change (FC) of LRRC19 was evaluated by log transformation. |logFC| > 1 and adjusted *P* < 0.01 were regarded as the screened threshold. Subsequently, the Venn software was used online (https://bioinformatics.psb.ugent.be/webtools/Venn/) to recognize the original data among the four datasets and to reveal the commonly expressed DEGs.

### 2.3. LRRC19 mRNA Expression Analysis across Oncomine and TIMER

The LRRC19 gene expression levels in various types of cancers were identified via the Oncomine database and the TIMER database. The summarization of LRRC19 expression in different tumor samples and its specific expression in CRC specimens compared with adjacent normal tissues were analyzed via the Oncomine portal (https://www.oncomine.org) [14]. The cutoff values were a *P* value of 0.01, fold change of 1.5, top 10% gene ranking, and the data had to be from mRNA. The fold change in LRRC19 expression in various datasets is given in Table 1. We also analyzed LRRC19 expression in different types of cancer via the Tumor IMmune Estimation Resource (TIMER) database (https://timer.cistrome.org) [15].

### 2.4. Patients and Tissue Specimens

Tumor tissues from 56 CRC cancer patients were collected between May 2020 and February 2021 at Shengzhou People's Hospital and subjected to quantitative reverse transcriptase PCR (qRT-PCR) analysis. Fifteen of these tissues had adjacent normal samples for control. Our experiments were in accordance with the ethical standards formulated in the Helsinki Declaration. This study was authorized by the Ethics Committee of Shengzhou People's Hospital Health Authority.

### 2.5. Quantitative Real-Time PCR (qRT-PCR)

Total RNA was extracted from CRC tissues using TRIzol reagent (ThermoFisher Scientific, USA). Subsequently, the cDNA was amplified by a Reverse Transcriptional Kit (Promega, USA). The real-time PCR was performed using SYBR Premix Ex Taq II (Takara Bio Inc., Otsu, Japan) by an ABI 7500 Fast Real-Time PCR System (ThermoFisher Scientific, USA). GAPDH was employed as the internal reference to control, and the mRNA level was evaluated using a threshold cycle value, for which the formula was 2^−ΔΔCT^, where ^Δ^CT = (CT (target gene)-CT (GAPDH)). The primers used were as follows: LRRC19: forward: 5′-ATGAAAGTCACAGGCATCACAATCC-3′ and reverse: 5′-ATTTTCTTCACATAATTCATGGATA-3′; and GAPDH: forward: 5′-TCGACAGTCAGCCGCATCTTCTTT-3′ and reverse: 5′-ACCAAATCCGTTGACTCCGACCTT-3′.

### 2.6. Immunohistochemistry

Immunohistochemistry was performed as previously described [16]. Anti-LRRC19 antibody was used to detect protein expression in myocardial tissue. The Olympus microscope was used to capture images at 200 magnification, and 3 fields of view of each sample were randomly selected to quantify the relative intensity of protein staining.

### 2.7. Survival Analysis in CRC via GEPIA and PrognoScan

The PrognoScan database (https://dna00.bio.kyutech.ac.jp/PrognoScan/index.html) was utilized to analyze the correlation between LRRC19 expression and survival in various types of cancers [17]. Associations between gene expression and patient prognosis, such as overall survival (OS) and disease-free survival (DFS), were searched on the PrognoScan site. The threshold was defined as a Cox *P* value <0.05. Subsequently, the correlation between LRRC19 expression and overall survival of CRC patients was explored on the GEPIA website (https://gepia.cancer-pku.cn/) using COAD-TCGA and READ-TCGA. GEPIA is a Web-based tool to deliver fast and customizable functionalities based on TCGA and GTEx data [18].

### 2.8. Survival Analysis in Patients with Malignancies through the Kaplan–Meier Plotter

The Kaplan–Meier Plotter (https://kmplot.com/analysis/) is an online tool containing the survival information (including OS, RFS, PPS, DMPS, FP, or PFS) of patients with breast cancer, lung cancer, gastric cancer, or ovarian cancer. It can estimate the effect of 54,675 genes on survival using 10,461 cancer samples. We used the Kaplan–Meier plotter to analyze the relationship between LRRC19 expression and survival in breast, ovarian, lung, and gastric cancers [19]. The log-rank *P* value and hazard ratio (HR) with 95% confidence intervals were also calculated.

### 2.9. GO and KEGG Pathway Enrichment Analysis

The GO (https://www.geneontology.org) database can provide functional classification for genomic data, including biological process (BP), cellular component (CC), and molecular function (MF) [20]. It is a widely used annotating tool of genes and gene products. The Kyoto Encyclopedia of Genes and Genomes (KEGG, https://www.genome.ad.jp/kegg/) database is a networked website designed for genic function analysis, annotation, and visualization [21]. In this study, GO enrichment analysis and KEGG pathway analysis were performed using the “enrichplot” package in *R* software (https://www.R-project.org/) to explore the biological functions of LRRC19. A *P* value <0.05 was considered statistically significant.

### 2.10. Gene Correlation Analysis of LRRC19

To identify other molecules that might have a relationship with LRRC19, protein-protein interaction analysis (PPI) was performed using the STRING database (https://string-db.org/). Then, we used the online database GEPIA for further gene expression correlation analysis, which was performed on The Cancer Genome Atlas (TCGA) expression data. Spearman's correlation analysis was conducted, the nonlog scale was used for calculation, and the log-scale axis was used for visualization.

### 2.11. Statistical Analysis

All data were analyzed using the SPSS statistical package (version 17.0; SPSS Inc., Chicago, IL, USA). The associations between LRRC19 expression and clinicopathological characteristics were evaluated by the chi-square tests and Spearman's correlation analysis. Student's *t*-test was performed to compare the expression of LRRC19 between CRC and adjacent normal tissues. Data were analyzed by GraphPad Prism software and presented as mean ± SD indicated in the figure legends. *P* < 0.05 was considered to denote a statistically significant difference.

## 3. Results

### 3.1. DEG Filtering

Four GSE datasets were obtained from the GEO database as follows: GSE21510, GSE22598, GSE23878, and GSE41328 (Table 2). Through analysis conducted using the GEO2R online tool with the cutoff criterion of adjusted *P* < 0.05 and |log2FC| ≥ 2, the results showed that GSE21510 consisted of 955 DEGs, GSE22598 included 342 DEGs, GSE23878 contained 481 DEGs, and GSE41328 contained 206 DEGs. Finally, the commonly expressed 67 DEGs, including 21 upregulated and 46 downregulated genes, were discovered in the CRC tissues compared with the paracarcinoma tissue by Venn software in the four datasets (Table 3, Figure 1(c), and Figures S1(A) and S1(B)). Of the above genes, we found that LRRC19 was one of the downregulated genes in CRC samples (Figure 1(b)) and its character in CRC was unclear. As a result, LRRC19 was ultimately selected for further study.

### 3.2. mRNA Expression Levels of LRRC19 in Different Types of Human Cancers

**Figure 2:**
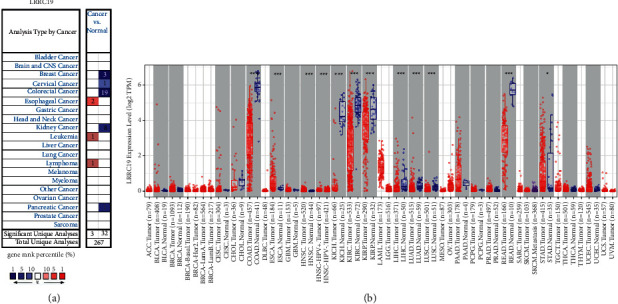
LRRC19 expression levels in different types of human cancers. (a) Increased or decreased LRRC19 in datasets of different cancers compared with normal tissues in the Oncomine database. (b) Human LRRC19 expression levels in different tumor types from the TCGA database determined by TIMER (^∗^*P* < 0.05, ^∗∗^*P* < 0.01, ^∗∗∗^*P* < 0.001).

The Oncomine database analysis proved that LRRC19 mRNA expression in CRC was reduced according to 19 of 23 analyses compared with the normal tissues (Figure 2(a) and Table 1). Additionally, LRRC19 mRNA expression was lower in breast cancer, cervical cancer, kidney cancer, and pancreatic cancer. Meanwhile, higher expression levels were observed in esophageal cancer, leukemia, and lymphoma in some datasets.

To further assess LRRC19 expression in human cancers, we detected LRRC19 expression using the TIMER database (Figure 2(b)). The discriminate expression between the tumor and adjacent normal tissues for LRRC19 across most tumors is shown in Figure 2(b). LRRC19 expression was obviously lower in colon adenocarcinoma (COAD), kidney chromophobe (KICH), kidney renal clear cell carcinoma (KIRC), kidney renal papillary cell carcinoma (KIRP), liver hepatocellular carcinoma (LIHC), lung adenocarcinoma (LUAD), lung squamous cell carcinoma (LUSC), and rectum adenocarcinoma (READ) compared with adjacent normal tissues. Nevertheless, LRRC19 expression was obviously higher in esophageal carcinoma (ESCA), head and neck cancer (HNSC), and stomach adenocarcinoma (STAD) compared with adjacent normal tissues.

### 3.3. Validation of the mRNA Expression Pattern of LRRC19 in CRC Clinical Samples

**Figure 3:**
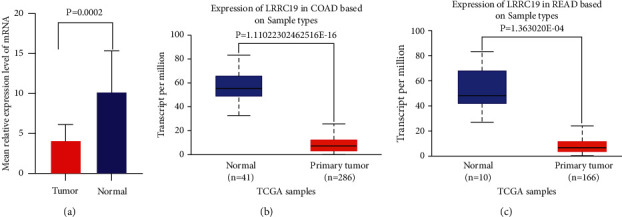
LRRC19 expression was decreased in colorectal cancer specimens at mRNA. (a) The average LRRC19 expression ± SD for all studied tumors and their corresponding normal tissues (*P* < 0.001). *Y*-axis, the mean relative expression level of LRRC19 expression normalized to normal tissues, GAPDH as an internal control. (b)-(c) The mRNA expression of LRRC19 analyzed using TCGA-COAD and TCGA-READ datasets through UALCAN website.

The aforementioned pancancer analysis of LRRC19 expression showed that it was significantly lower in CRC tissues compared with adjacent normal tissues. To further confirm the distinguishing LRRC19 mRNA expression in patients after radical resection for CRC, we performed qRT-PCR on 15 paired CRC and noncancerous colorectal tissues (Figure S1(C)). The results further confirmed the significantly lower expression of LRRC19 mRNA in CRC, compared with adjacent normal tissues (*P* < 0.001, Figure 3(a)). The result of qRT-PCR was further confirmed through the UALCAN website (Figures 3(b) and 3(c)) (for subtype analysis, Figure S2), which is an interactive Web portal for analyzing cancer transcriptome data based on the TCGA gene expression data [22].

### 3.4. Validation of the Immunohistochemistry of LRRC19 in CRC Clinical Samples

**Figure 4:**
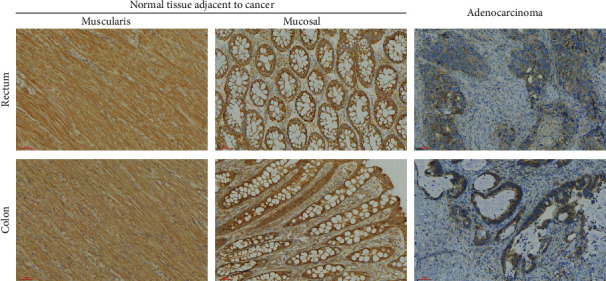
LRRC19 expression decreased in colorectal cancer specimens at protein. Expression levels of LRRC19 protein in adenocarcinoma samples from patients with colorectal cancer (rectum, *n* = 9) and colorectal cancer (colon, *n* = 9) and in adjacent normal tissues (muscularis, mucosal) (200x).

Furthermore, we collected tumor samples from patients with colorectal cancer and normal tissues adjacent to the tumor for pathological sections and used immunohistochemistry to evaluate the expression of LRRC19 in colorectal cancer. As a result, as shown in Figure 4, we could establish that LRRC19 expression was also significantly reduced in CRC samples compared with adjacent normal control tissues. This indicated that LRRC19 was also significantly decreased in human CRC tissue at the protein function level.

### 3.5. Correlation of LRRC19 Expression with the Clinicopathological Factors of CRC

The LRRC19 mRNA levels in 56 CRC tissues were further correlated with the clinicopathological characteristics of CRC (Table 4). Based on the average value of LRRC19 mRNA level, there were 30 patients with high LRRC19 expression and 26 patients with low LRRC19 expression. LRRC19 expression was negatively associated with *T* stage (*P*=0.038) and *N* stage (*P*=0.047). Meanwhile, no important correlation was discovered between LRRC19 expression and other clinicopathological features, including age (*P*=0.589), gender (*P*=0.278), tumor location (*P*=0.399), pathology stage (*P*=0.104), and *M* stage (*P*=0.211).

### 3.6. Association of Lower LRRC19 Expression with the Shorter Survival of CRC Patients

**Figure 5:**
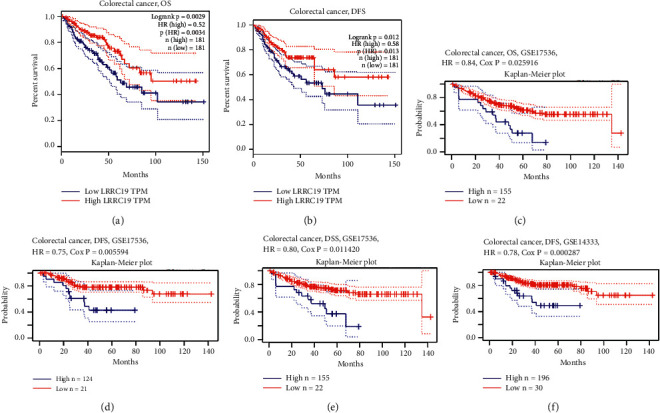
Correlation between LRRC19 expression and survival of CRC patients. (a)-(b) Survival curves of OS and DFS in the database of TCGA through GEIPA website. (c)–(f) Kaplan–Meier survival curves of OS, DSS, and DFS compared the high and low expressions of LRRC19 in two colorectal cancer cohorts (GSE17536 (*n* = 177) and GSE14333 (*n* = 226)) on the PrognoScan website. OS, overall survival; DFS, disease-free survival; DSS, disease-specific survival.

We used the TCGA database through the GEIPA website to study the correlation between LRRC19 mRNA expression and the survival of patients with CRC (data based on COAD and READ modules). The results showed that low levels of LRRC19 mRNA expression in CRC tissues were significantly correlated with poorer overall survival (OS) (*P* < 0.059) among patients with CRC (Figures 5(a) and 5(b)). Subsequently, LRRC19 expression was assessed by means of the PrognoScan website and was remarkably found to significantly affect the prognosis of CRC patients. Two cohorts (GSE17536 and GSE14333) (30, 31) containing 226 specimens at different stages of CRC proved that low LRRC19 expression was markedly related to poorer prognosis (OS HR = 0.84, 95% CI = 0.72 to 0.98, Cox *P*=0.025916; DFS HR = 0.75, 95% CI = 0.61 to 0.92, Cox *P*=0.005594; DSS HR = 0.80, 95% CI = 0.68 to 0.95, Cox *P*=0.011420; DFS HR = 0.78, 95% CI = 0.68 to 0.89, Cox *P*=0.000287) (Figures 5(c)–5(f)). Thereby, it can be conjectured that low LRRC19 expression is an independent risk factor and indicates poor prognosis in CRC patients. The lower the expression of LRRC19, the worse the shorter survival of CRC patients.

### 3.7. Lower LRRC19 Expression Displays Poorer Prognosis of Patients with Malignancies

**Figure 6:**
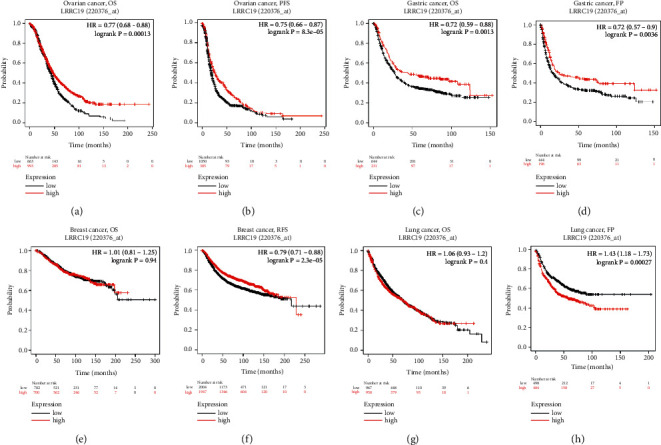
Kaplan–Meier survival curves comparing the high and low expressions of LRRC19 in different types of cancer in the Kaplan–Meier plotter databases. (a)-(b) OS and PFS survival curves of ovarian cancer (*n* = 1,657, *n* = 1,436). (c)-(d) OS and FP survival curves of gastric cancer (*n* = 881, *n* = 645). (e)-(f) OS and RFS survival curves of breast cancer (*n* = 1,402, *n* = 3,955). (g)-(h) OS and FP survival curves of lung cancer (*n* = 1,927, *n* = 982). OS, overall survival; PFS, progression-free survival; FP, first progression; RFS, relapse-free survival.

To further evaluate the prognostic potential of LRRC19 in different cancers, we used the Kaplan–Meier plotter database to evaluate the LRRC19 prognostic significance in four types of cancer, including breast cancer, ovarian cancer, lung cancer, and gastric cancer (Figure 6). Interestingly, poor prognosis in ovarian cancer (OS HR = 0.77, 95% CI = 0.68 to 0.88, *P*=0.00013; PFS HR = 0.75, 95% CI = 0.66 to 0.87, *P*=8.3*e* − 05) and gastric cancer (OS HR = 0.72, 95% CI = 0.59 to 0.88, *P*=0.0013; FP HR = 0.72, 95% CI = 0.57 to 0.9, *P*=0.0036) was shown to correlate with lower LRRC19 expression (Figures 6(a)–6(d)). Meanwhile, low LRRC19 expression was correlated with poor prognosis of RFS in BRCA and had less influence on OS (Figures 6(e) and 6(f)). However, low LRRC19 expression was correlated with a better prognosis of FP in lung cancer (Figures 6(g) and 6(h)). These results revealed that lower LRRC19 was significantly associated with the prognosis of patients with malignancies, including breast cancer, lung cancer, gastric cancer, and ovarian cancer. In addition, increased or decreased LRRC19 expression has different prognostic effects according to the cancer type.

### 3.8. Gene Ontology Function Enrichment Analysis of LRRC19 in CRC

**Figure 7:**
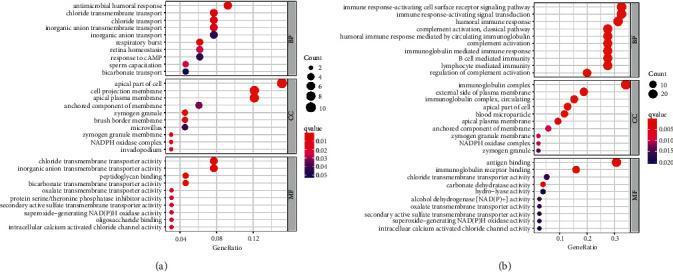
Top 10 GO term enrichment analysis of LRRC19 in CRC. (a) LRRC19-related BP, CC, and MF analyses in COAD. (b) LRRC19-related BP, CC, and MF analyses in READ. GO, Gene Ontology; BP, biological process; CC, cellular component; MF, molecular function.

To identify the biological significance and function of LRRC19, we performed an enrichment analysis for LRRC19 (Figure 7). The GO enrichment analysis showed the functional roles of LRRC19 in three ways, including biological process (BP), cellular components (CC), and molecular function (MF). For biological processes, our results revealed that LRRC19 was largely involved in antimicrobial humoral response, chloride transmembrane transport, chloride transport, inorganic anion transmembrane transport, and inorganic anion transport in COAD. Meanwhile, in READ, LRRC19 mainly took part in immune response-activating cell surface receptor signaling pathway, immune response-activating signal transduction, humoral immune response, complement activation, classical pathway, humoral immune response mediated by circulating immunoglobulin, complement activation, immunoglobulin-mediated immune response, B-cell-mediated immunity, and lymphocyte-mediated immunity. For cellular components, both COAD and READ revealed that downregulated LRRC19 was associated with apical part of cell, apical plasma membrane, anchored component of membrane, zymogen granule, NADPH oxidase complex, and zymogen granule membrane. For molecular function, downregulated LRRC19 was principally enriched in chloride transmembrane transporter activity, inorganic anion transmembrane transporter activity, protein serine/threonine phosphatase inhibitor activity, superoxide-generating NAD(P)H oxidase activity, and intracellular calcium-activated chloride channel activity in COAD. At the same time, LRRC19 was mainly enriched in antigen binding, immunoglobulin receptor binding, chloride transmembrane transporter activity, superoxide-generating NAD(P)H oxidase activity, and intracellular calcium-activated chloride channel activity in READ (for immune subtype, Figure S3).

### 3.9. KEGG Pathway Enrichment Analysis of LRRC19 in CRC

**Figure 8:**
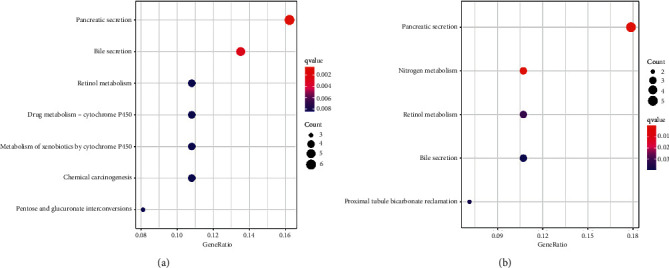
KEGG pathway enrichment analysis of LRRC19 in CRC. (a) LRRC19-related pathways in COAD. (b) LRRC19-related pathways in READ. KEGG, Kyoto Encyclopedia of Genes and Genomes.

The KEGG pathway analysis revealed that LRRC19 was enriched in pancreatic secretion, bile secretion, pentose and glucuronate interconversions, drug metabolism-cytochrome P450, retinol metabolism, metabolism of xenobiotics by cytochrome P450, and chemical carcinogenesis in COAD (Figure 8(a)). In READ, we revealed that LRRC19 was enriched in pancreatic secretion, nitrogen metabolism, retinol metabolism, bile secretion, and proximal tubule bicarbonate reclamation (Figure 8(b)). Remarkably, our data support that LRRC19 plays an important role by regulating the above cancer-related signaling pathways.

### 3.10. Forecast and Explanation of Associations between LRRC19 and Other Molecular Networks

**Figure 9:**
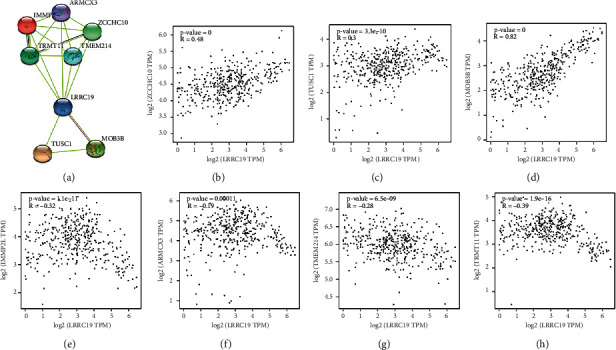
Network prediction and annotation of LRRC19 with other molecules. (a) Seven candidate molecules might have an interaction with LRRC19. (b)–(h) The GEPIA database showed that the above candidate molecules might be associated with LRRC19. (b)–(d) Positive relation (*R* > 0). (e)–(h) Negative relation (*R* < 0).

To determine the other molecules that might have an association with LRRC19, we applied an interaction network prediction using the STRING database (https://string-db.org) and further evaluation in the GEPIA database to increase the authenticity of the result (Figure 9). According to the results, a total of 7 candidate proteins (ARMCX3, IMMP2L, MOB3B, TRMT11, TUSC1, ZCCHC10, and TMEM214) might have an interaction with LRRC19 in this study (Figure 9(a)). Afterwards, we sought for the relationship between LRRC19 and 7 molecules in the GEPIA database. We used the non-log scale for calculation and the log-scale axis for visualization. The results indicated that the estimates for both database sites were partially congruent (Figures 9(b)–9(h)). We found that ZCCHC10 and MOB3B were positively correlated with LRRC19 (Pearson's correlation coefficient: *R* > 0.3; Figures 9(b) and 9(d)), whereas IMMP2L and TRMT11 were negatively correlated with LRRC19 (Pearson's correlation coefficient *R* < −0.3; Figures 9(e) and 9(h)), but there is no obvious linear relationship between TUSC1, ARMCX3, and TMEM214 with LRRC19 based on the current data (−0.3 ≤ *R* ≤ 0.3; Figures 9(c), 9(f), and 9(g)).

## 4. Discussion

During the past several decades, dietary, lifestyle, inflammatory infection, medication risk factors, and genetics have been confirmed to be involved in the generation and progression of CRC; however, the detailed molecular mechanism remains unclear [23]. Even though important advancements have been made in terms of looking into the underlying mechanisms associated with the diagnosis, treatment, and prognosis estimation of CRC, more optimal and new CRC biomarkers are yet in urgent need. Thus, in our study, we utilized bioinformatic tools to analyze four GEO datasets to screen more effective proto-oncogenes or tumor suppressors. We picked out LRRC19 as a potential tumor suppressor gene for its significant downregulation of mRNA levels in CRC tissues, as well as its largely unknown status in most of the tumors including CRC. More importantly, compared with previous studies, we further screened potential hub genes [24] that play important biological functions in CRC by adding an important dataset (GSE21510, which has the most CRC samples).

LRRC19, a functional transmembrane receptor, belongs to the mammalian protein subgroup of singleton LRR-only group within the LRR family [25, 26]. Except for LRRC19, the mammalian protein subgroup also contains 14 kinds of leucine-rich repeat (LRR) proteins, such as LRRC17, LRRC25, and Lrg1. In postmenopausal women, the low plasma level of LRRC17 was considered as an independent risk factor for osteoporotic fractures [27]. In addition, in ovarian cancer, LRRC17 also acts as a prognostic gene as it regulates cancer cell viability via the p53 pathway [28]. Hoffman et al. proposed that LRRC25 might increase the risk of breast cancer, given that elevated LRRC25 leads to an enhanced inflammatory response [29]. Zhang and colleagues found that overexpressed LRG1 could induce the EMT process and angiogenesis in colorectal cancer [30]. However, only few studies have been conducted on the LRRC19 in human tumors. This study showed that the LRRC19 protein appears to be specifically expressed in the kidney, spleen, and intestine [31]. Liu et al. reported that the reduced expression of LRRC19 was an independent risk factor for OS and LRRC19 could serve as a novel biomarker for prognosis and adjuvant treatment of selenium [32]. Chai et al. proposed that LRRC19 can activate NF-kB and induce the expression of pro-inflammatory cytokines and is involved in the response to local inflammation [31]. Cao et al. reported that LRRC19 is associated with enteritis, colitis, and colitis-associated tumorigenesis in Lrrc19 KO mice [33]. LRRC19 was also mentioned for its therapeutic potential in pressure ulcers, by promoting NF-kB-dependent pro-inflammatory response [34]. The latest research demonstrated that LRRC19 can increase the permeability of the gut epithelial barrier by degrading PKC to reduce the expression of ZO-1, ZO-3, and occludin [35].

In our study, we first evaluated LRRC19 mRNA expression in human CRC using data from the TIMER and Oncomine online tools. The results displayed that LRRC19 mRNA expression was dramatically reduced in CRC tissues compared with adjacent normal tissues, which was further verified by the qPCR result for paired clinical samples. The above results of LRRC19 differential expression were immediately confirmed by the UALCAN website. These findings revealed that the expression of LRRC19 mRNA was significantly decreased in CRC tissues, which might be necessary for the occurrence and progression of CRC. Subsequently, we also investigated the association between LRRC19 mRNA with the clinicopathological characteristics in CRC patients. LRRC19 expression was negatively related to *T* stage (*P*=0.038) and *N* stage (*P*=0.047) and showed no significant difference in age, gender, tumor location, pathology stage, and *M* stage between the two groups. In addition, we adopted the GEPIA and PrognoScan websites to assess the prognostic value of LRRC19 expression. Our research revealed that reduced LRRC19 expression was markedly associated with shorter OS, DFS, or DSS in CRC patients. These data remind us that downregulated LRRC19 might be a general event in CRC and a beneficial biomarker for the prognosis of CRC patients. Further survival analysis in four human common cancers (including breast cancer, lung cancer, gastric cancer, and ovarian cancer) inferred that lower LRRC19 expression significantly correlates with poorer progression (including OS, RFS, PPS, DMPS, FP, or PFS) in patients, suggesting the potential value of lower LRRC19 expression on prognostic prediction.

To further clarify the biological functions of LRRC19 in CRC, GO and KEGG pathway analyses were carried out (Figures 7 and 8). Overall, our analysis suggested that low expression of LRRC19 could be mainly involved in the transmembrane transport, immune response, NADPH oxidase complex, zymogen granule membrane, and retinol metabolism, leading to the tumorigenesis and progression of CRC. Among the identified pathways, retinol metabolism pathway took part in CRC occurrence, and the incidence of CRC was associated with lower serum levels of retinol [36]. Of the screened biological processes, the immune response has also been found to be implicated in colorectal cancer [37, 38], which offers us a new research direction of anticancer therapeutic approach via immune checkpoint inhibition. Chai et al. also verified that LRRC19 can be involved in the response to local inflammation [31]. For cellular components, NADPH oxidase and zymogen granule have long been recognized as essential factors in colorectal carcinogenesis and development. For instance, upregulated NOX4 predicts poor prognosis and facilitates tumor progression in CRC [39]. Meng et al. proved that zymogen granule protein 16 (ZG16) regulates PD-L1 expression and immune response in CRC [40]. Furthermore, LRRC19 is involved in a number of molecular functions related to tumor progression, such as protein serine/threonine phosphatase inhibitor activity and superoxide-generating NAD(P)H oxidase activity. The above study indicates that LRRC19 participates in many biological processes involving tumorigenesis and progression.

However, through GO and KEGG enrichment analyses, we also found some interesting points. It can be seen that in terms of BP and MF in the GO enrichment analysis, in COAD, the function of LRRC19 is more closely related to some ion transport; in READ, the relationship between LRRC19 and immune activation is stronger. However, in the CC plate, the functions performed by LRRC19 are relatively close. We speculate that, at the functional level, LRRC19 has different biases in COAD and READ, which may be related to their specific locations, but LRRC19, as a functional transmembrane receptor, naturally has greater similarities in cellular components. However, when several types of molecules with different functional tendencies are enriched in the KEGG pathway, it can be seen that their main functions are similar in COAD and READ, which is also in line with the characteristics of LRRC19 transmembrane transporter, which is related to the secretion of various substances, metabolic hooks.

Finally, to discern the other molecules that might interact with LRRC19, we carried out a network prediction of LRRC19 with other molecules in the STRING database along with further validation on the GEPIA website. We found that LRRC19 might interact with ZCCHC10, MOB3B, IMMP2L, and TRMT11. Some of these known or predicted interacted genes had been reported to be oncogenes or tumor suppressor genes. For example, for zinc finger CCHC-type-containing 10 (ZCCHC10), acting as a direct target of miR-410-3p in CRC, miR-410-3p-mediated ZCCHC10 suppression can facilitate the EMT process, cell migration, and invasion of CRC cells by means of regulating NF-*κ*B activation [41]. ZCCHC10 can also inhibit lung cancer progression and cisplatin resistance through reducing MDM2-mediated p53 ubiquitination and degradation [42]. These shreds of evidence underline that ZCCHC10 plays its role as a tumor suppressor gene in CRC and lung cancer.

Meanwhile, ZCCHC10, which may have a positive linear relationship with LRRC19, can reduce the ubiquitination and degradation of p53 and inhibit EMT and cell migration. MASPIN, which is also closely related to p53 in CRC, can also inhibit tumor proliferation and has antiangiogenic and proapoptotic properties. Coincidentally, MASPIN is a useful tool for identifying tumor buds and, through examining the subcellular localization of its staining, for evaluating EMT in CRC. Cytoplasmic MASPIN positivity is associated with the best prognosis, but nuclear MASPIN positivity is associated with the shortest survival time and high invasion [43, 44]. Therefore, we speculate that there may be a connection between LRRC19 and MASPIN, and this connection may make LRRC19 assist MASPIN in the molecular classification of CRC. However, the biological function and detailed molecular mechanism of LRRC19 in CRC should be further handled in future studies.

All in all, our research has certain novel and exciting findings, which may help other researchers in the field or scholars interested in this direction. For example, the low expression of LRRC19 may be mainly involved in biological processes such as transmembrane transport and immune response, which can provide the possibility of in-depth research for the majority of scholars. Second, we screened several molecular markers that may be directly related to LRRC19 by combining STRING and GEPIA databases, which also provide a bridge for further exploration of the detailed molecular mechanism of LRRC19.

## 5. Conclusion

Overall, this study is the first to demonstrate that LRRC19 prominently decreases in human CRC. Moreover, reduced LRRC19 expression was significantly associated with shorter survival in CRC patients. The results of the functional enrichment pathway and PPI analyses suggest that LRRC19 might be a tumor suppressor gene. Additional research will be needed to seek the detailed mechanisms of LRRC19 function and its possible clinical value in CRC.

## Figures and Tables

**Table 1:** Oncomine analysis of LRRC19 mRNA expression in colorectal cancer.

Cohort no.	Cohort	Microarray	Sample (*n*)	Fold change	*P* value
1	Hong colorectal	mRNA	Colorectal carcinoma vs. normal	−12.606	1.87*E* − 32

2	Skrzypczak colorectal 2	mRNA	Colon carcinoma epithelia vs. normal	−4.523	5.01*E* − 11
		mRNA	Colon adenoma epithelia vs. normal	−3.598	5.64*E* − 09
		mRNA	Colon carcinoma vs. normal	−5.626	1.02*E* − 08
		mRNA	Colon adenoma vs. normal	−6.367	8.55*E* − 07

3	Kaiser colon	mRNA	Colon mucinous adenocarcinoma vs. normal	−10.541	3.22*E* − 08
		mRNA	Cecum adenocarcinoma vs. normal	−5.335	8.25*E* − 08
		mRNA	Colon adenocarcinoma vs. normal	−4.466	5.5*E* − 10
		mRNA	Rectal adenocarcinoma vs. normal	−5.807	0.0000303
		mRNA	Rectosigmoid adenocarcinoma vs. normal	−6.676	0.0000224

4	Gaedeke colorectal	mRNA	Rectal adenocarcinoma vs. normal	−7.798	3.04*E* − 35

5	Skrzypczak colorectal	mRNA	Colorectal adenocarcinoma vs. normal	−2.757	3.71*E* − 14
		mRNA	Colorectal carcinoma vs. normal	−4.481	1.74*E* − 10

6	Sabates–Bellver colon	mRNA	Colon adenoma vs. normal	−2.58	5.24*E* − 13
		mRNA	Rectal adenoma vs. normal	−2.955	0.000167

7	TCGA colorectal	mRNA	Colon mucinous adenocarcinoma vs. normal	−11.699	2.11*E* − 13
		mRNA	Rectal adenocarcinoma vs. normal	−7.875	8.12*E* − 15
		mRNA	Cecum adenocarcinoma vs. normal	−8.857	4.47*E* − 10
		mRNA	Colon adenocarcinoma vs. normal	−6.529	3.42*E* − 13

**Table 2:** Detailed information of the four GEO datasets.

ID	Contributor(s), year	Tumor	Nontumor	Platform
GSE21510	Tsukamoto et al., 2010	104	44	Affymetrix Human Genome U133 Plus 2.0 Array

GSE22598	Okazaki et al., 2010	17	17	Affymetrix Human Genome U133 Plus 2.0 Array

GSE23878	Uddin et al., 2010	35	24	Affymetrix Human Genome U133 Plus 2.0 Array

GSE41328	Lin et al., 2012	10	10	Affymetrix Human Genome U133 Plus 2.0 Array

**Table 3:** All 67 commonly differentially expressed genes (DEGs).

DEGs	Total	Gene name
Upregulated	21	CDH3, MMP7, TRIB3, FOXQ1, MMP3, INHBA, NFE2L3, CEMIP, AZGP1, CLDN2, CXCL8, DPEP1, ASCL2, AJUBA, CLDN1, EPHX4, COL11A1, CTHRC1, MMP1, CRNDE, KRT23

Downregulated	46	LGALS2, NR3C2, SPIB, HSD17B2, ABCG2, ZG16, GUCA2B, CHP2, SCARA5, CLCA4, DHRS11, AKR1B10, ARL14, CA4, NXPE4, SCIN, TSPAN7, CA2, FCGBP, PKIB, ANPEP, CEACAM7, ABCA8, MUC2, BEST2, SLC51B, ADH1B, AQP8, GCG, CD177, MS4A12, PCK1, ADH1C, HEPACAM2, UGT2A3, GCNT2, LRRC19, SCNN1B, C2orf88, LAMA1, BEST4, CA1, SI, GUCA2A, DHRS9, CA7

**Table 4:** Association between LRRC19 mRNA and clinicopathological factors in CRC patients.

Characteristics	Total (*N* = 56)	LRRC19 mRNA		*P* value
		Low (*n* = 26)	High (*n* = 30)	
Age				0.589
≤65	22	9	13	
>65	34	17	17	

Gender				0.278
Male	33	13	20	
Female	23	13	10	

Tumor location				0.399
Colon	19	7	12	
Rectum	37	19	18	

Pathology stage				0.104
I	0	0	0	
II	29	9	20	
III	27	15	12	

*T* stage				0.038
T1	1	0	1	
T2	9	2	7	
T3	31	13	18	
T4	15	11	4	

*N* stage				0.047
N0	34	12	22	
N1	12	6	6	
N2	10	8	2	

*M* stage				0.211
M0	54	24	30	
M1	2	2	0	

## Data Availability

The data used to support this study are mainly based on public databases and are available after the remaining part of the experimental data is verified.

## Supplementary Materials

Supplementary Materials Figure S1: (A) Venn diagram reveals all 67 DEGs in the four datasets (|log2FC| ≥ 2). (B) 21 DEGs upregulated in the four datasets (log2FC ≥ 2). (C) LRRC19 mRNA expression in 15 pairs of CRC tissues and noncancerous colorectal tissues measured by qPCR analysis. Figure S2: mRNA expression of LRRC19 analyzed using TCGA-COAD and TCGA-READ datasets through UALCAN website and a full TCGA subtype analysis. Figure S3: correlation between LRRC19 gene expression and TILs in COAD and READ and MESO by the TIMER database.
