# Supplementary figures and images for: Downregulation of LRRC19 Is Associated with Poor Prognosis in Colorectal Cancer
Source: J Oncol. 2022 Jun 26;2022:5848823. doi: 10.1155/2022/5848823 (PMC9251150; doi:10.1155/2022/5848823)

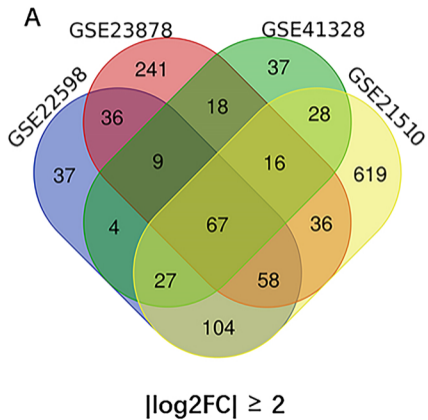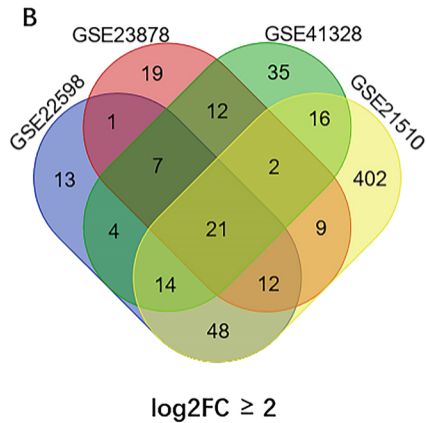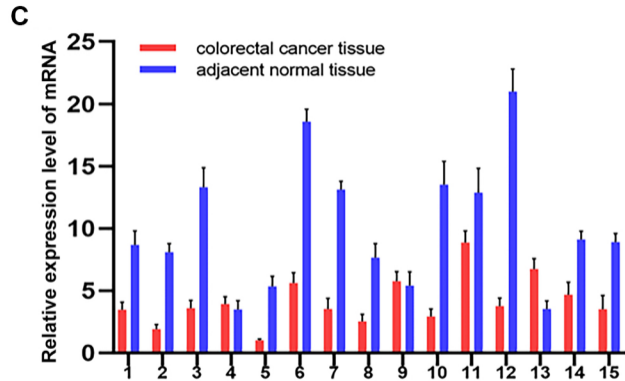

Supplement: Supplementary Materials — Figure S1: (A) Venn diagram reveals all 67 DEGs in the four datasets (|log2FC| ≥ 2). (B) 21 DEGs upregulated in the four datasets (log2FC ≥ 2). (C) LRRC19 mRNA expression in 15 pairs of CRC tissues and noncancerous colorectal tissues measured by qPCR analysis. Figure S2: mRNA expression of LRRC19 analyzed using TCGA-COAD and TCGA-READ datasets through UALCAN website and a full TCGA subtype analysis. Figure S3: correlation between LRRC19 gene expression and TILs in COAD and READ and MESO by the TIMER database. [file 5848823.f1.zip › 5848823.f1/Figure supply 1.pdf]

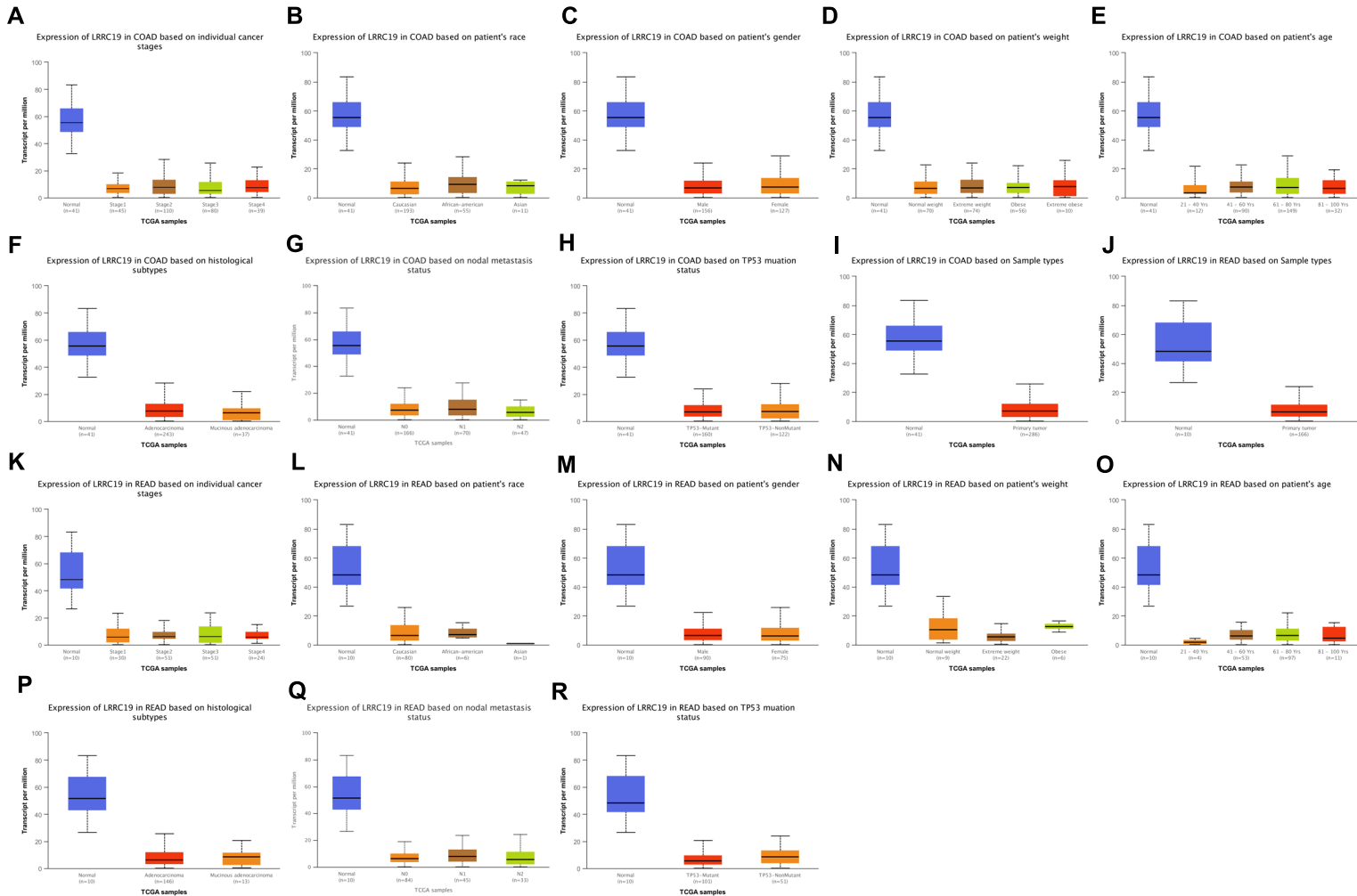

Supplement: Supplementary Materials — Figure S1: (A) Venn diagram reveals all 67 DEGs in the four datasets (|log2FC| ≥ 2). (B) 21 DEGs upregulated in the four datasets (log2FC ≥ 2). (C) LRRC19 mRNA expression in 15 pairs of CRC tissues and noncancerous colorectal tissues measured by qPCR analysis. Figure S2: mRNA expression of LRRC19 analyzed using TCGA-COAD and TCGA-READ datasets through UALCAN website and a full TCGA subtype analysis. Figure S3: correlation between LRRC19 gene expression and TILs in COAD and READ and MESO by the TIMER database. [file 5848823.f1.zip › 5848823.f1/Figure supply 2.pdf]

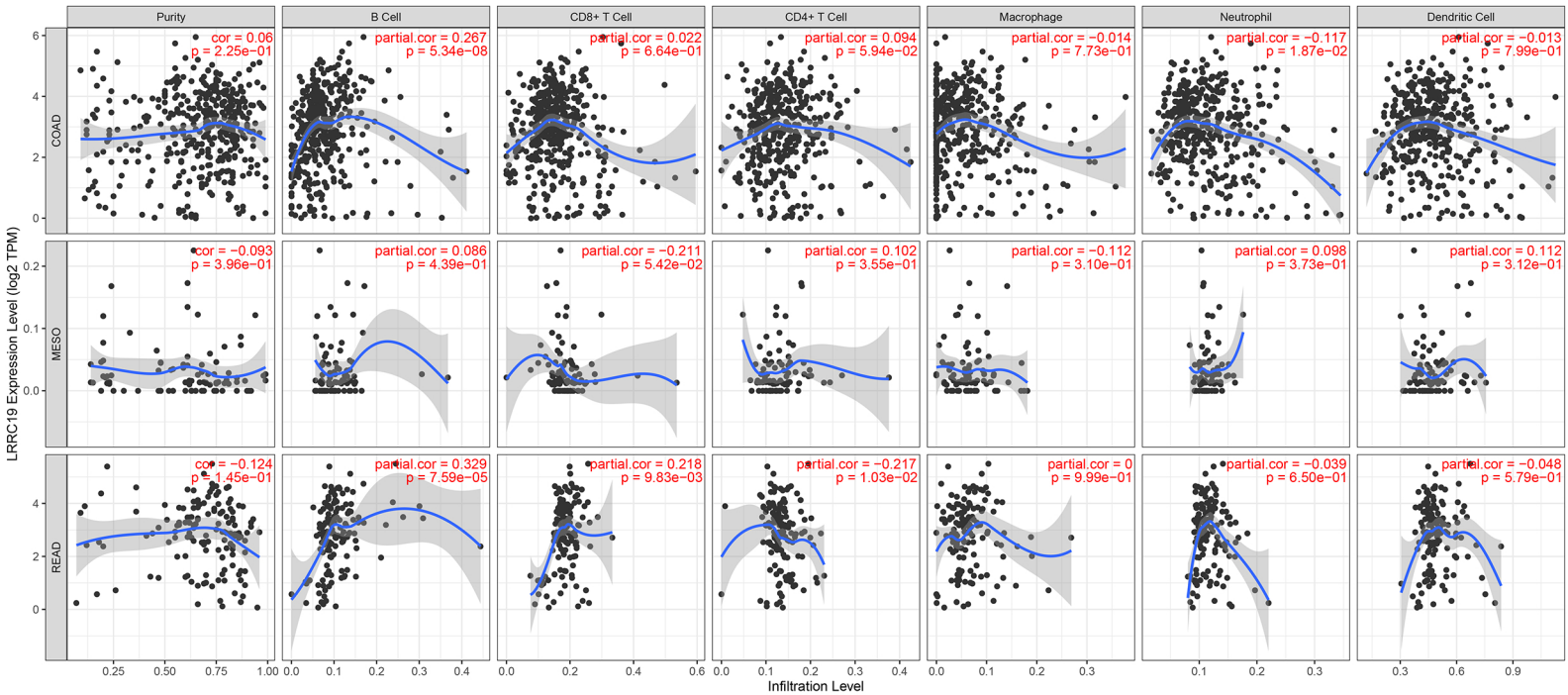

Supplement: Supplementary Materials — Figure S1: (A) Venn diagram reveals all 67 DEGs in the four datasets (|log2FC| ≥ 2). (B) 21 DEGs upregulated in the four datasets (log2FC ≥ 2). (C) LRRC19 mRNA expression in 15 pairs of CRC tissues and noncancerous colorectal tissues measured by qPCR analysis. Figure S2: mRNA expression of LRRC19 analyzed using TCGA-COAD and TCGA-READ datasets through UALCAN website and a full TCGA subtype analysis. Figure S3: correlation between LRRC19 gene expression and TILs in COAD and READ and MESO by the TIMER database. [file 5848823.f1.zip › 5848823.f1/Figure supply 3.pdf]
